# Effectiveness of Titanium Occlusive Barriers in Guided Bone Regeneration: A Prospective Analysis of Vertical and Horizontal Bone Augmentation

**DOI:** 10.3390/biomimetics10030165

**Published:** 2025-03-07

**Authors:** Luis Leiva-Gea, Paulino Sánchez-Palomino, Alfonso Lendínez-Jurado, María Daniela Corte-Torres, Isabel Leiva-Gea, Antonio Leiva-Gea

**Affiliations:** 1 Clínicas ClearDent, 23006 Jaén, Spain; 2 Facultad de Odontología, Universidad de Murcia, 30003 Murcia, Spain; 3 Facultad de Odontología, Universidad de Granada, 18011 Granada, Spain; 4 Hospital Regional Universitario de Málaga, 29011 Málaga, Spain; 5 Facultad de Medicina, Universidad de Málaga, Andalucía Tech, Campus de Teatinos s/n, 29071 Málaga, Spain; 6 Instituto de Investigación Biomédica de Málaga (IBIMA)-Plataforma BIONAND, 29010 Málaga, Spain; 7 Biobanco del Principado de Asturias, FINBA-ISPA, 33011 Oviedo, Spain; 8 Hospital Universitario Virgen de la Victoria, 29010 Málaga, Spain

**Keywords:** guided bone regeneration, titanium barriers, bone augmentation, dental implants

## Abstract

Background: Guided bone regeneration (GBR) is a widely used technique in oral and maxillofacial surgery to restore lost bone. The aim of this study is to evaluate the effectiveness of titanium occlusive barriers in GBR for increasing bone volume in both vertical and horizontal dimensions. Methods: A prospective analysis was conducted on 11 patients (15 cases) undergoing bone augmentation with titanium barriers combined with bone graft biomaterials for dental implant placement. Bone gain was assessed using pre- and postoperative low-dose cone beam computed tomography (CBCT) measurements in vertical and horizontal planes. Histological analyses evaluated the quality and vascularization of the regenerated bone. Results: Significant bone volume increases were observed, with a mean vertical gain of 7.60 mm (SD 0.23) and a horizontal gain of 5.44 mm (SD 0.39). Histological examination confirmed well-vascularized regenerated bone with minimal residual graft material, effective integration, and the formation of keratinized gingiva. Conclusions: Titanium occlusive barriers in GBR provide a reliable and minimally invasive method for substantial bone regeneration, showing advantages such as ease of handling and reduced invasiveness. Additional studies are recommended to validate these findings and evaluate long-term outcomes.

## 1. Introduction

The use of oral implants is often compromised by bone resorption that occurs following tooth loss. Within the first few months after losing a tooth, both horizontal and vertical bone reduction is observed, which can continue up to five years later [1,2]. Alveolar preservation during extractions can minimize bone resorption, facilitating implant placement and increasing its success rate [3,4].

Currently, there are various guided bone regeneration (GBR) techniques that have proven effective in promoting both bone height and width [5,6]. The presence of keratinized gingiva around the bone is crucial to prevent marginal bone loss and ensure the durability of the implant [7]. However, neither GBR nor alveolar preservation can completely prevent tissue changes, often necessitating additional soft tissue grafts [8].

Digitalization in GBR offers greater precision and predictability, facilitating the surgeon’s work and reducing complications [9,10,11,12,13,14]. The use of titanium occlusive barriers seems to be an effective tool for GBR, requiring appropriate management to minimize complications and optimize clinical outcomes. However, little evidence supporting the technique has been found up to now [15,16].

The combination of autogenous blood with titanium barriers in GBR appears to be as successful as the use of bioceramic graft materials [17]. The blood clot provides a scaffold for cell adhesion, which orchestrates subsequent alveolar healing [18,19]. The use of tricalcium phosphate as a biomaterial has been described in this technique to facilitate osteoconduction. The purpose of the filling material is to stabilize the fibrin mesh of the clot, preserving the clot matrix, which will be invaded by vascular cells, leading to the initial granulation of osteoid tissue. For bone tissue formation, a vascular stroma is essential to allow the invasion of osteogenic cells [20,21].

Conventional guided bone regeneration (GBR) techniques pose significant challenges, such as the need for autologous bone harvesting, which increases morbidity and the risk of complications, including wound dehiscence.

The success of GBR largely hinges on adherence to the four fundamental principles of PASS: primary wound closure, angiogenesis, space creation, and wound stability. Strict compliance with these principles is essential for the successful execution of GBR techniques and for achieving optimal outcomes [22].

This study proposes the use of titanium occlusive barriers using GBR for the subsequent osseointegration of the implant. A series of cases is presented that describe the surgical technique and analyze the variable outcomes of vertical and horizontal bone gain.

## 2. Materials and Methods

### 2.1. Study Design

This prospective cohort study included patients with alveolar bone defects treated with custom-made titanium barriers for guided bone regeneration. Written informed consent was obtained from all participants, followed by an initial cone beam computed tomography (CBCT) scan to assess the bone defect. Based on the CBCT images, a custom titanium barrier was designed and placed over the defect, remaining in situ for six months. After barrier removal, a follow-up CBCT scan was performed two months later (eight months after the initial surgery) to evaluate bone regeneration. Finally, at ten months (four months post-barrier removal), implants were placed, and a biopsy was collected for histological analysis.

Implementation and reporting of the study were based on STROBE cohort reporting guidelines.

### 2.2. Patient Recruitment

Patient recruitment occurred between October 2022 and August 2023, involving 11 patients and a total of 15 cases.

### 2.3. Inclusion Criteria

Adults over 18 years of age with alveolar bone defects classified as type 2/4 or type 3/4 according to Terheyden’s classification [23].

### 2.4. Exclusion Criteria

Patients with diabetes, hypertension, or other uncontrolled systemic diseases, as well as pregnant women, were excluded from the study.

### 2.5. Ethics

The study protocol was in accordance with the Declaration of Helsinki. It was approved by the Research Ethics Committee of Jaén (1794-N-22). Informed consent was obtained from all patients for both surgical procedure and their inclusion in the study.

### 2.6. Preoperative Information

Prior to the regenerative surgery, necessary information was collected through medical history, oral photographs using a camera, intraoral scanning (PRISMECAN^®^), and low-dose cone beam CT (CBCT).

CBCT images were acquired in the Digital Imaging and Communications in Medicine (DICOM^®^) format and processed using Simpleware ScanIP software version 7.0. The generated three-dimensional mask was exported as a stereolithography (.STL) file. This STL file was imported into Rhinoceros design software version 6.0, where the Osteophoenix^®^ titanium barrier was designed in three dimensions. The barrier was manufactured from Ti6Al4V via laser sintering at approximately 350 °C (660 °F) with a porosity of 30–60 microns. The barrier had a thickness of 0.7 mm, with length customized for each individual case. Subsequently, pink anodization was performed, followed by milling using CAM software Magics 23, sintering at 1450 °C, and sterilization in an autoclave (TINHERO-16 Class B, Runyes) at 134 °C.

**Figure 1:**
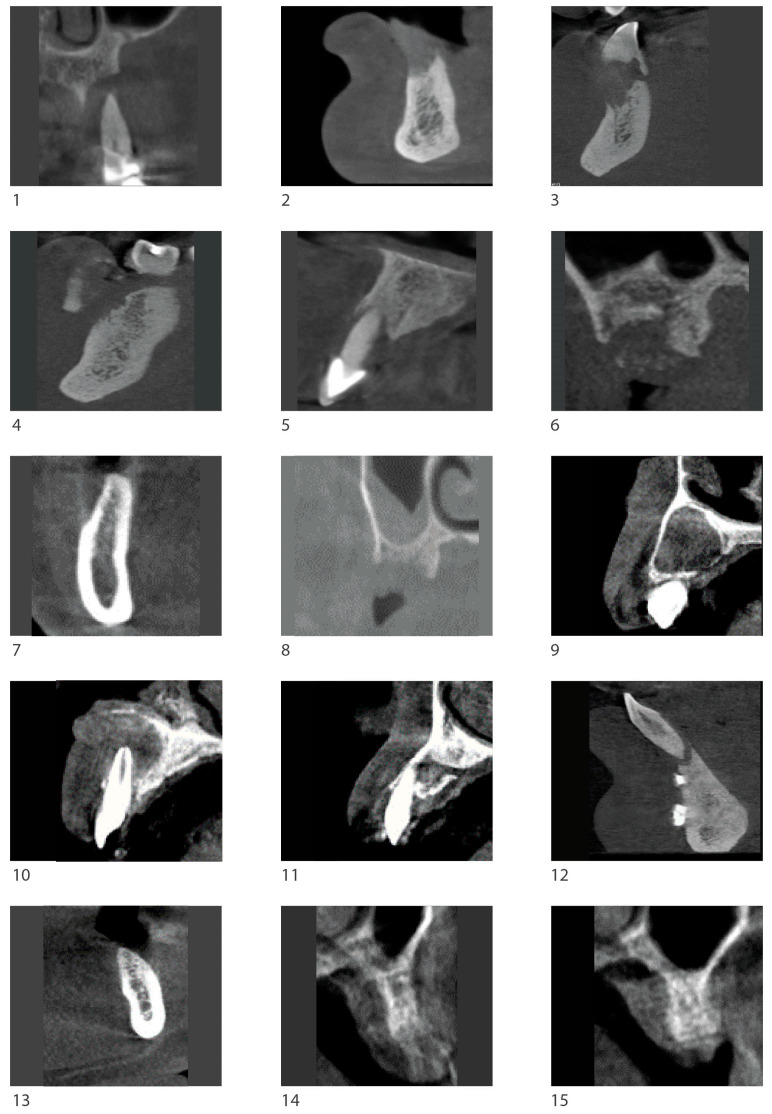
Sagittal pre-surgical CBCT images showing the residual atrophic area before surgery. Numbers 1–15 represent the 15 cases described, nested within 11 patients. Using the tooth numbering system for identification, the location of each case is described as follows: Case 1: Tooth 25, Case 2: Tooth 42, Case 3: Tooth 33, Case 4: Tooth 34, Case 5: Tooth 21, Case 6: Tooth 17, Case 7: Tooth 46, Case 8: Tooth 26, Case 9: Tooth 13, Case 10: Tooth 11, Case 11: Tooth 13, Case 12: Tooth 41, Case 13: Tooth 36, Case 14: Tooth 25, Case 15: Tooth 26.

Figure 1 shows CBCT images of the cases.

**Figure 2:**
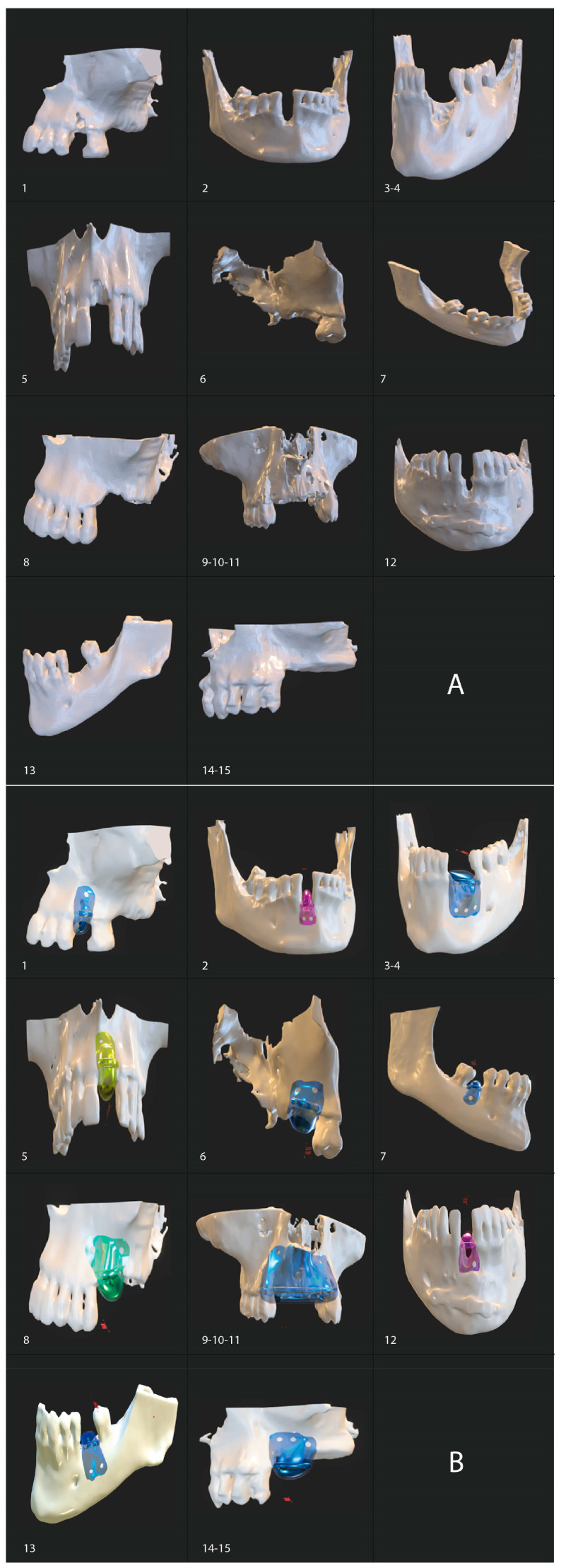
(**A**) Virtual design of the bone defects and fabrication of biomodels using stereolithography. (**B**) Virtual design of the barriers in the defects. Numbers 1–15 represent the 15 cases described, nested within 11 patients. Using the tooth numbering system for identification, the location of each case is described as follows: Case 1: Tooth 25, Case 2: Tooth 42, Case 3: Tooth 33, Case 4: Tooth 34, Case 5: Tooth 21, Case 6: Tooth 17, Case 7: Tooth 46, Case 8: Tooth 26, Case 9: Tooth 13, Case 10: Tooth 11, Case 11: Tooth 13, Case 12: Tooth 41, Case 13: Tooth 36, Case 14: Tooth 25, Case 15: Tooth 26.

Figure 2 shows images of the virtual design of the bone defects and the biomodels fabricated via stereolithography, as well as the virtual design of the barriers in the defects.

### 2.7. Surgical Protocol

#### 2.7.1. Barrier Placement

**Figure 3:**
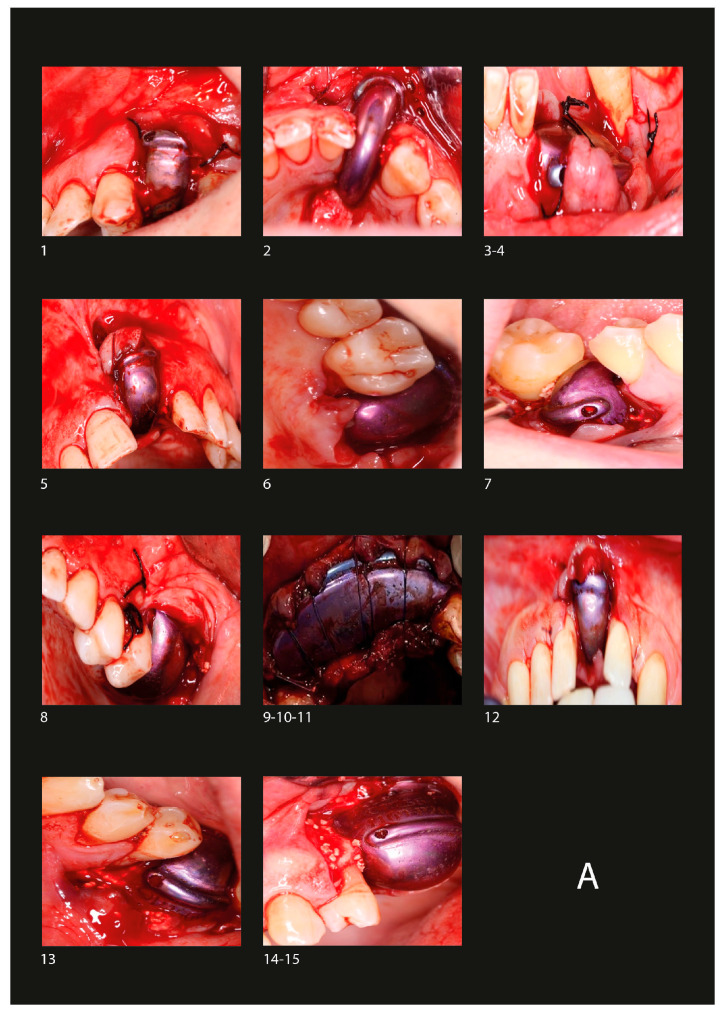

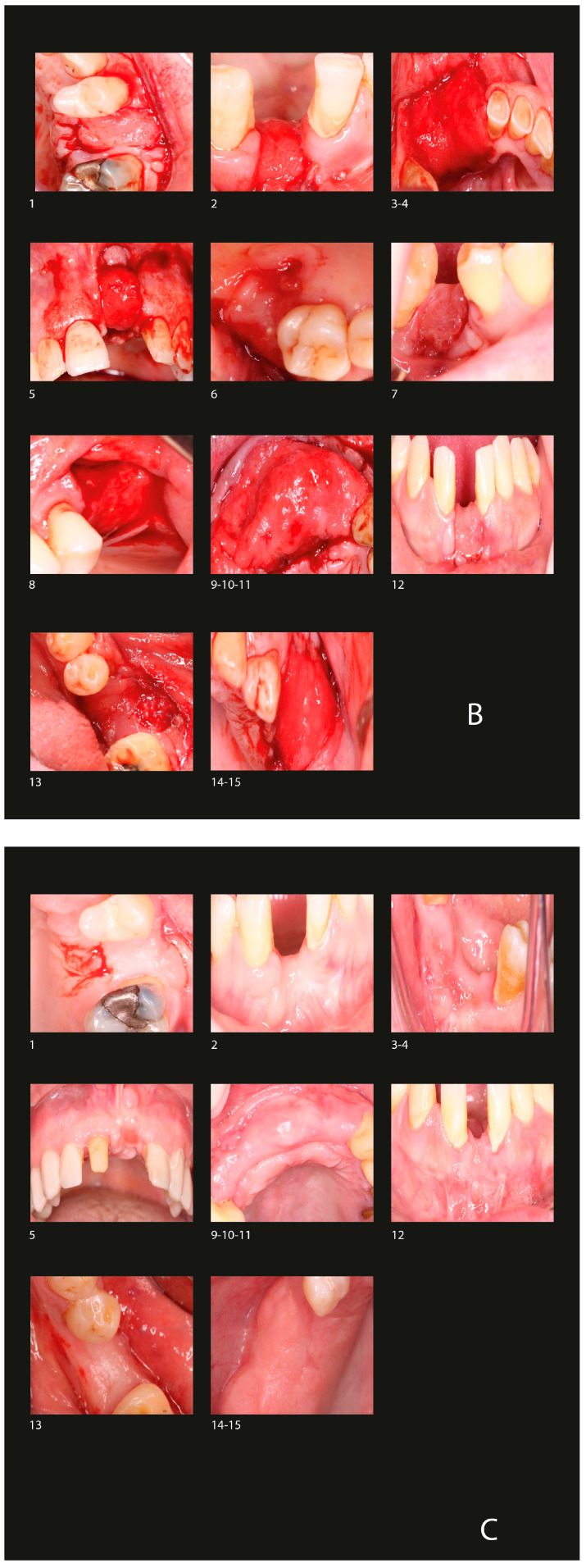
(**A**) Placement of the barriers in the area for GBR. (**B**) Removal of the barriers. (**C**) Placement of the implants in the GBR area. Numbers 1–15 represent the 15 cases described, nested within 11 patients. Using the tooth numbering system for identification, the location of each case is described as follows: Case 1: Tooth 25, Case 2: Tooth 42, Case 3: Tooth 33, Case 4: Tooth 34, Case 5: Tooth 21, Case 6: Tooth 17, Case 7: Tooth 46, Case 8: Tooth 26, Case 9: Tooth 13, Case 10: Tooth 11, Case 11: Tooth 13, Case 12: Tooth 41, Case 13: Tooth 36, Case 14: Tooth 25, Case 15: Tooth 26.

Before surgery, blood was drawn from the patient to be used for clot formation. Local infiltrative anesthesia with 4% articaine was administered in the surgical area. The surgical incision design was considered to include the highest bone peak within the barrier, making incisions at the papilla with subsequent elevation of a full-thickness soft tissue flap to expose the alveolar bone, if necessary. Decortication was performed to promote angiogenesis [24]. The blood clot was fragmented and mixed with tricalcium phosphate [20]. The prepared clot was transferred to the regeneration area and secured with 1.50 mm diameter titanium screws, using a 1.30 mm drill at 450 rpm with sterile saline solution (SSS). Finally, the incision area was nonabsorbable 3-0 sutured with simple interrupted sutures and some approximation sutures at the crestal incision (Figure 3A).

#### 2.7.2. Postoperative Care

After surgery, antibiotic therapy was initiated with amoxicillin–clavulanic acid or clindamycin in case of allergy, along with prescribed analgesics for one week.

At 14 days post-operation, irrigation with SSS was performed through a predesigned aperture in the barrier. This irrigation was continued weekly for 6 months at home. Starting from the first month, the initial irrigation was performed with 0.5% hydrogen peroxide mixed with SSS, followed by subsequent irrigations with SSS alone.

The barrier was removed 6 months after the initial surgery (Figure 3B).

#### 2.7.3. Implant Placement

Implant placement was performed 4 months after barrier removal (Figure 3C).

Surgical guides were used to avoid disturbing potentially immature tissues. Tomographic assessments were subsequently conducted to verify the correct positioning of the implants.

### 2.8. Variables Analyzed

A. Relationship with bone and soft tissue regeneration

 1. Radiographic Measurements

CBCT scans of the entire arch were taken 21 days before surgery and 8 months afterward. CT data were exported in DICOM format. The DICOM data were analyzed using 3DSlicer medical software version 5.2.1 (https://slicer.org accessed on 7 February 2023) [25]. Preoperative and postoperative studies were segmented using the software’s thresholding tool, and the resulting segments were superimposed. The difference between the preoperative and postoperative segments was re-segmented to obtain the graft segment, which was then exported in STL format [26]. The assessment of image was conducted by two observers and mean values were reported.

 2. Histological analysis

**Figure 4:**
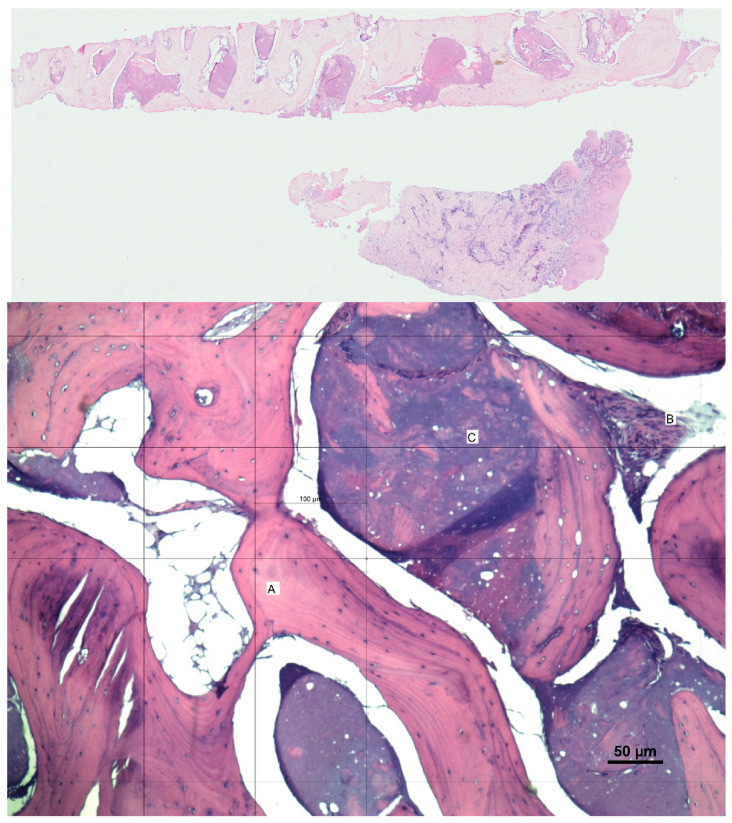
This figure illustrates Case 3. Medullary bone (trabecular or spongy) with predominantly laminar trabeculae (**A**), separated by intertrabecular spaces filled with basophilic amorphous material (**C**) and dense, richly vascular fibrous stroma (**B**). Quantitative analysis revealed that trabecular bone constituted 72% of the examined section, with a notable density of osteocytes and active osteoblasts, indicating ongoing bone formation.

In the moment of implant placement, 4 months after barrier removal, a trephine was used to drill to the desired implant depth, and the obtained sample was fixed in 10% formalin solution. Tissues were embedded in paraffin, sectioned at 5-micron thickness, and stained with hematoxylin and eosin for histological analysis to determine the degree of bone formation (Figure 4).

B. Relationship with procedure. Complications

 1. Fixation loss

Defined as the difference in the position of the titanium barrier relative to its initial location.

 2. Local infections

### 2.9. Statistical Analysis

Data analysis was conducted utilizing R version 4.4.2 (R Core Team, 2020), an open-source statistical software package (available at https://www.r-project.org/, accessed on 7 February 2023). Normality of the study variables was assessed using the Shapiro–Wilk test. Continuous data are expressed as mean ± standard deviation (SD) for normally distributed variables or as median (interquartile range (IQR)) for non-normally distributed variables. Comparative analyses were performed using the paired T-test for normally distributed data and the Wilcoxon signed-rank test for non-normally distributed data. Correlation analyses were conducted using Pearson’s correlation coefficient for parametric variables and Spearman’s correlation coefficient for nonparametric variables. Categorical variables were evaluated using the chi-square test. A significance threshold of *p* < 0.05 was applied for all statistical tests. To account for multiple comparisons, *p*-values were adjusted using the Benjamini–Hochberg correction.

## 3. Results

The mean age of the 11 patients (63.63% men) included in the study was 50.16 years, with a minimum age of 27 and a maximum age of 73 years.

### 3.1. Radiological Observation

**Figure 5:**
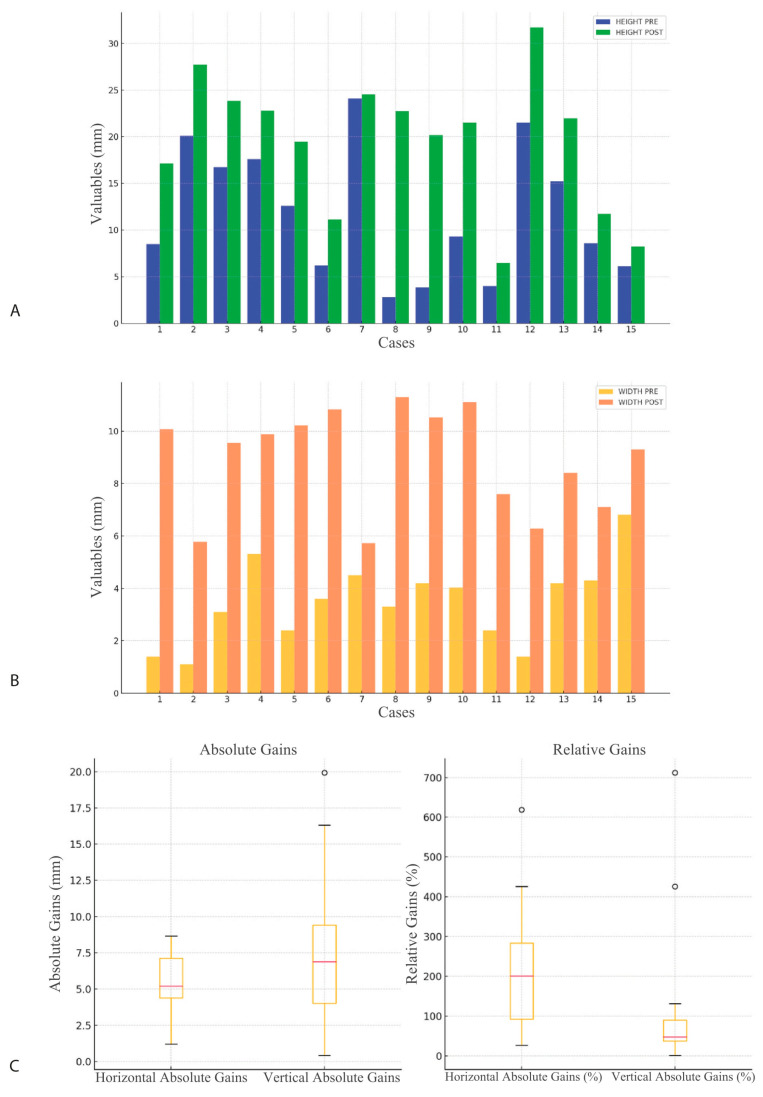
(**A**) Histogram for graphical evaluation of pre- and post-measurements of vertical and horizontal distances in mm. (**B**) Histogram for graphical evaluation of pre- and post-measurements of horizontal and vertical distances in mm. (**C**) Absolute and relative gains in horizontal and vertical distances.

The variables followed a normal distribution after applying the Shapiro–Wilk test. Paired sample *t*-tests revealed significant differences when comparing pre- versus post-operative measurements for both vertical and horizontal dimensions (Table 1) (Figure 5).

The absolute horizontal gain had a mean of 5.44 mm (SD 0.39), and the absolute vertical gain had a mean of 7.60 mm (SD 0.23). The relative horizontal gain was 215.14%, and the relative vertical gain was 122.79%.

### 3.2. Histological Observation

Histological examination revealed the presence of trabecular bone with a predominantly lamellar structure, indicating advanced bone formation. Residual graft material was observed in the samples, suggesting that a significant amount of new bone had formed around these remnants. Similarly, keratinized gingiva was shown. The presence of blood vessels in the regenerated bone further confirmed that the tissue was well vascularized, which is crucial for the viability and integration of the bone. Figure 4 shows the histological findings of Case 3. Other histological images corresponding to other cases are shown in Appendix A.

### 3.3. Complications

Two complications were observed, both associated with the loss of barrier fixation (Cases 7 and 13). A screw replacement was performed, achieving successful re-fixation and favorable outcomes. No local infections were evidenced during the follow-up period.

## 4. Discussion

One of the limitations of most GBR techniques is the need to harvest autologous bone from a donor site, which increases patient morbidity and the possibility of exposure of the grafted bone recipient area or exposed membrane [27]. The technique presented in this article does not require harvesting autologous bone from a donor site, as sufficient autologous bone is regenerated through the use of the patient’s blood clot combined with tricalcium phosphate.

The presented barrier technique does not require such rigorous soft tissue management since primary closure is not needed. Most GBR techniques involving a membrane or vertical ridge augmentation with a bone block are complex and technique-dependent interventions, where optimal management of soft and hard tissues is essential to avoid short- and long-term complications, requiring greater surgeon skill [27]. The proximity to muscle insertions and the lack of keratinized mucosa are factors that affect flap mobilization and, therefore, increase the risk of dehiscence [28]. These issues are not a limitation of the technique presented in this article, as muscle insertions do not need to be mobilized, nor is there a need for extensive soft tissue management. The four fundamental PASS principles (primary wound closure, angiosperm stability, space creation, stability of the wound) for the successful development of GBR techniques have been described [22,29]. The technique presented in this article allows for the omission of one of the PASS principles, which is primary closure, as soft tissue is not necessary for barrier placement.

To our knowledge, the technique involving the Osteophoenix^®^ occlusive titanium barrier has been described in only one publication [15] and has not been previously evaluated in any comparative studies with other regenerative techniques.

In this study, occlusive titanium barriers combined with GBR demonstrated a significant mean gain in both planes, with a mean vertical gain of 7.60 mm and a mean horizontal gain of 5.44 mm. These results indicate a considerable improvement in bone volume, far exceeding the initial values.

GBR with the urban technique has reported vertical bone gains of approximately 5 to 6 mm and horizontal gains of 4 to 5 mm, depending on the specific clinical situation [30]. Compared to occlusive titanium barriers, the urban technique shows similar results in the horizontal plane but slightly lower results in the vertical plane. This may be attributable to the lesser rigidity of reinforced membranes compared to titanium barriers, which may partially collapse if not properly handled. One factor that may maximize vertical gain is the inclusion of three cases from the same patient with alveolar cysts (Cases 9, 10 and 11).

On the other hand, the Khoury technique is highly effective for large and critical defects, providing substantial bone gain in both dimensions [31]. Khoury et al. have reported horizontal bone gains ranging from 4 to 7 mm and vertical gains from 5 to 8 mm. Compared to occlusive titanium barriers, the Khoury technique tends to provide similar or slightly superior vertical bone regeneration, with great bone stability due to the nature of the autologous bone graft. However, the Khoury technique is significantly more invasive and depends on the availability of sufficient autologous bone, which may limit its applicability in patients with low donor bone availability. Occlusive titanium barriers offer a less invasive alternative compared to the Khoury technique, with comparable results in terms of vertical and horizontal bone regeneration. Each technique has its own indications and limitations. The choice of technique should be based on the specific characteristics of the bone defect, the patient’s condition, and the surgeon’s experience.

Attending to the variables analyzed, radiological assessment may limit the evaluation of bone gain results, as immature bone characteristics may not be identified, and the presence of certain devices may alter radiological interpretation. These factors highlight the need for histological analysis to provide a more comprehensive evaluation.

The histological findings of this study underscore the effectiveness of the graft material in facilitating robust bone regeneration and vascular integration, providing a solid foundation for future dental procedures. The histological analysis revealed gingiva mucosa covered by squamous epithelium, indicating the presence of keratinized gingiva (Figure 4). The universal need for keratinized gingiva to improve peri-implant health is well known [32,33]. Most GBR techniques involve the modification of soft tissue position, necessitating a second surgery to achieve keratinized gingiva to ensure implant success. This limitation is minimized with the technique presented, but quantitative and qualitative studies are needed to delve deeper into these histological results, as well as to explore their correlation with other variables such as age or sex.

Compared to conventional techniques described in the literature, titanium barriers offer advantages in terms of less invasiveness and operational simplicity, as they do not require autologous bone harvesting and facilitate easier soft tissue management. However, additional studies are needed to directly compare this technique with others to assess its relative efficacy and determine its potential long-term benefits.

Limitations were found highlighting the small number of patients and short follow-up [34].

## 5. Conclusions

This prospective study aimed to evaluate the efficacy of titanium barriers in guided bone regeneration (GBR) for increasing bone volume in both vertical and horizontal dimensions. The significant results obtained, with a mean vertical gain of 7.60 mm and a horizontal gain of 5.44 mm, along with histological examination confirming the formation of well-vascularized bone and the presence of keratinized gingiva, underscore the effectiveness of titanium barriers as a useful and minimally invasive technique for substantial bone regeneration. These findings not only demonstrate the capability of the method but also highlight additional advantages, such as ease of handling and reduced invasiveness.

The contributions of this study lie in its potential to facilitate GBR practices through more efficient and less invasive techniques. However, limitations are acknowledged, including sample size and variability in application procedures, which may influence the observed outcomes. Further studies are recommended to validate these findings and assess long-term results, which could contribute to the standardization of procedures and improve clinical outcomes in future GBR applications.

## Figures and Tables

**Table 1:** Measurements in mm of vertical distance (height) and horizontal distance (width) by both observers with statistical analysis for mean comparison using paired *t*-tests.

Observer	Measurement	Pre (mm)	Post (mm)	*p*-Value
Observer 1	Width	3.47 (1.57)	8.91 (1.95)	0.0000
Observer 1	Height	11.81 (6.97)	19.41 (7.21)	0.0001
Observer 2	Width	3.47 (1.57)	8.84 (1.98)	0.0000
Observer 2	Height	11.77 (6.86)	19.34 (7.13)	0.0001

## Data Availability

The data supporting this study are openly available in the Redcap data repository at the Biomedical Research Institute of Malaga (IBIMA).

## Abbreviations

CBCT	cone beam computed tomography
GBR	guided bone regeneration
STL	stereolithography
SSS	sterile saline solution
DICOM	Digital Imaging and Communication in Medicine
SD	standard deviation
IQR	interquartile range

## Appendix A

Case 1. Spongy bone tissue.

Case 1. Periosteal surface with osteoblasts.

Case 2. Neoformed bone tissue, with mature, mineralized, viable spongy bone, housing intertrabecular spaces occupied by fibrous tissue.

Case 9. Area of intense osteoblastic activity.

Case 12. Osteoblastic and osteoclastic activity.

Case 12. Vascularized lacuna.
